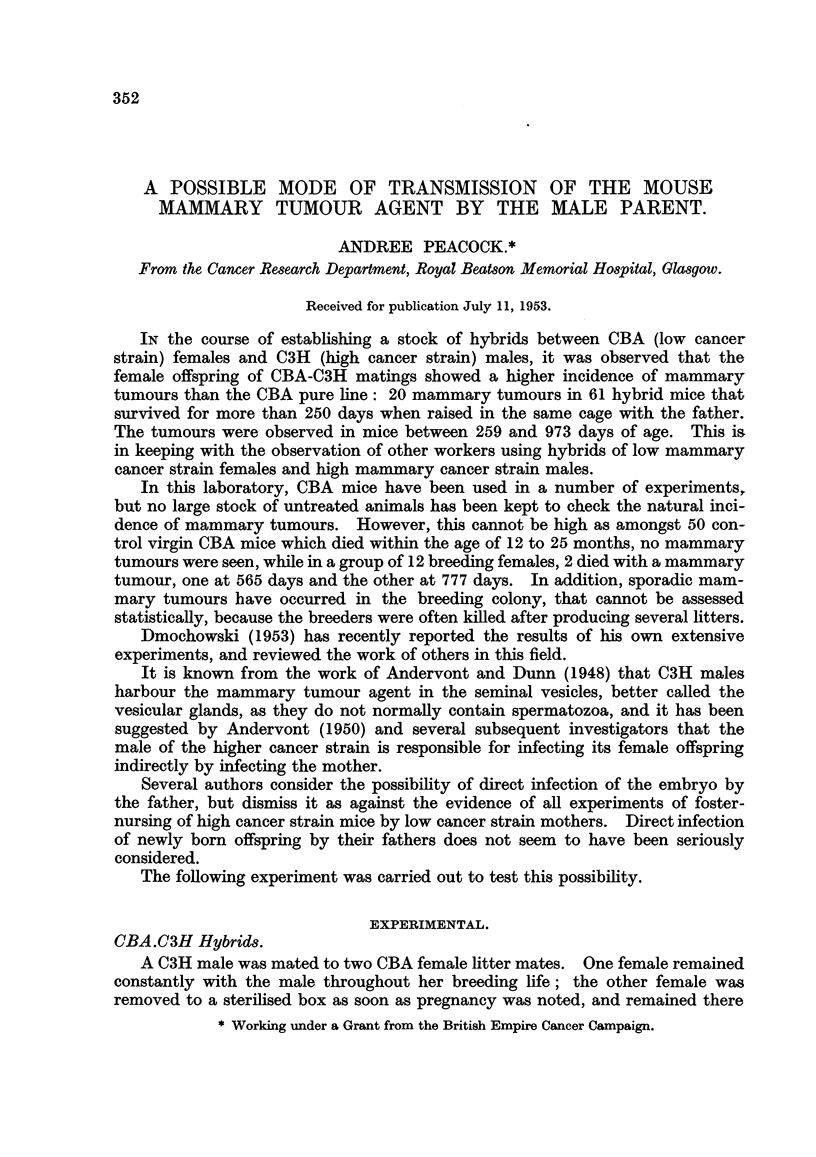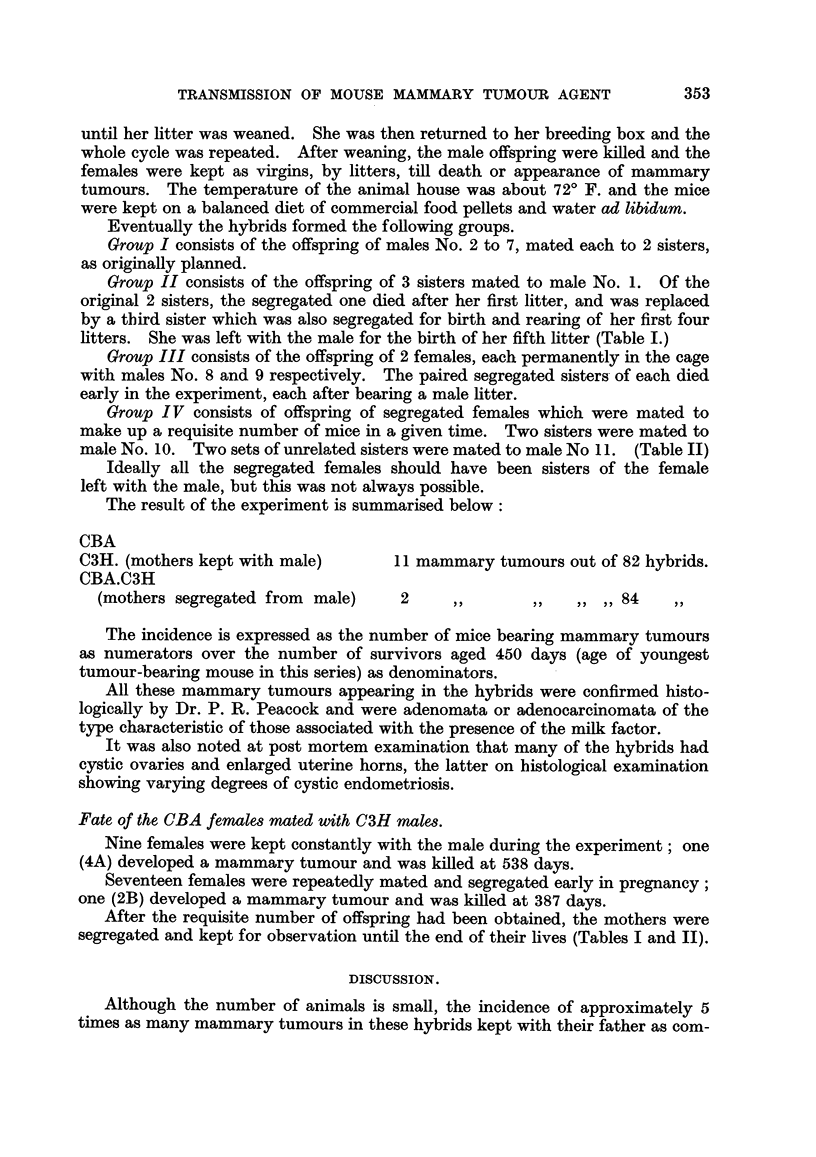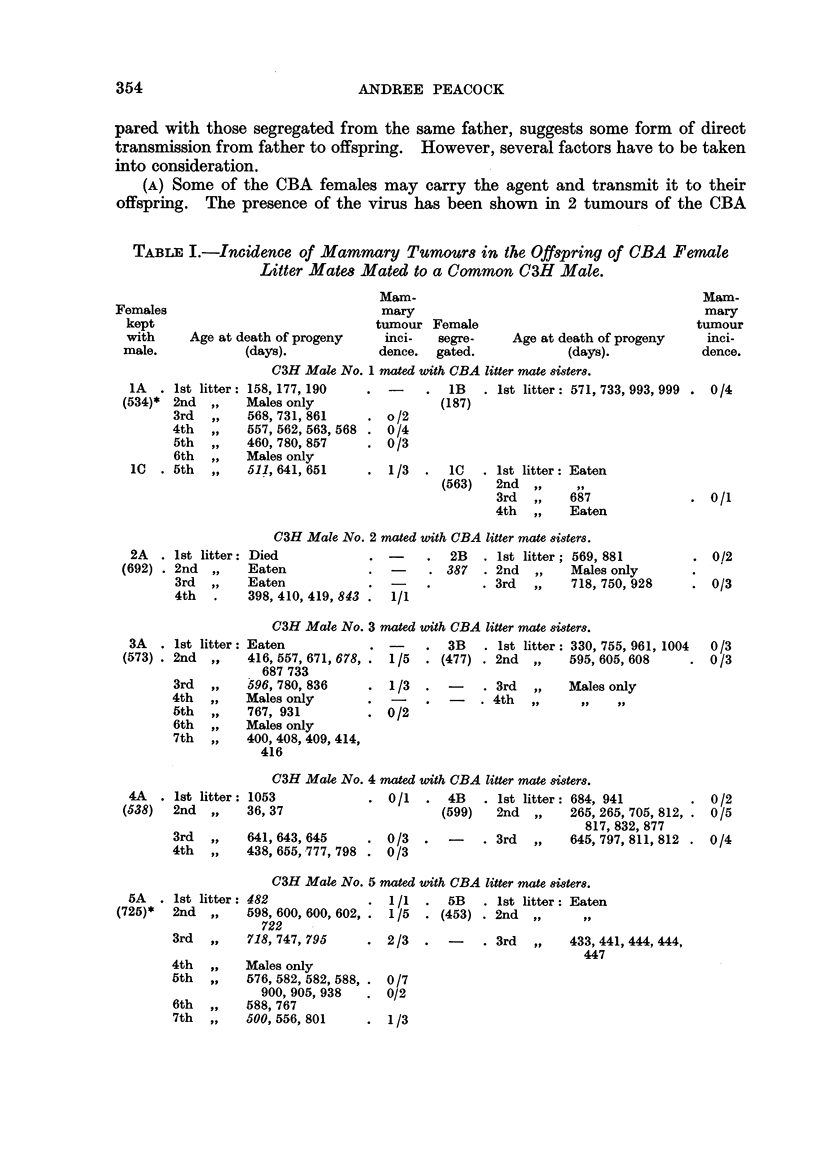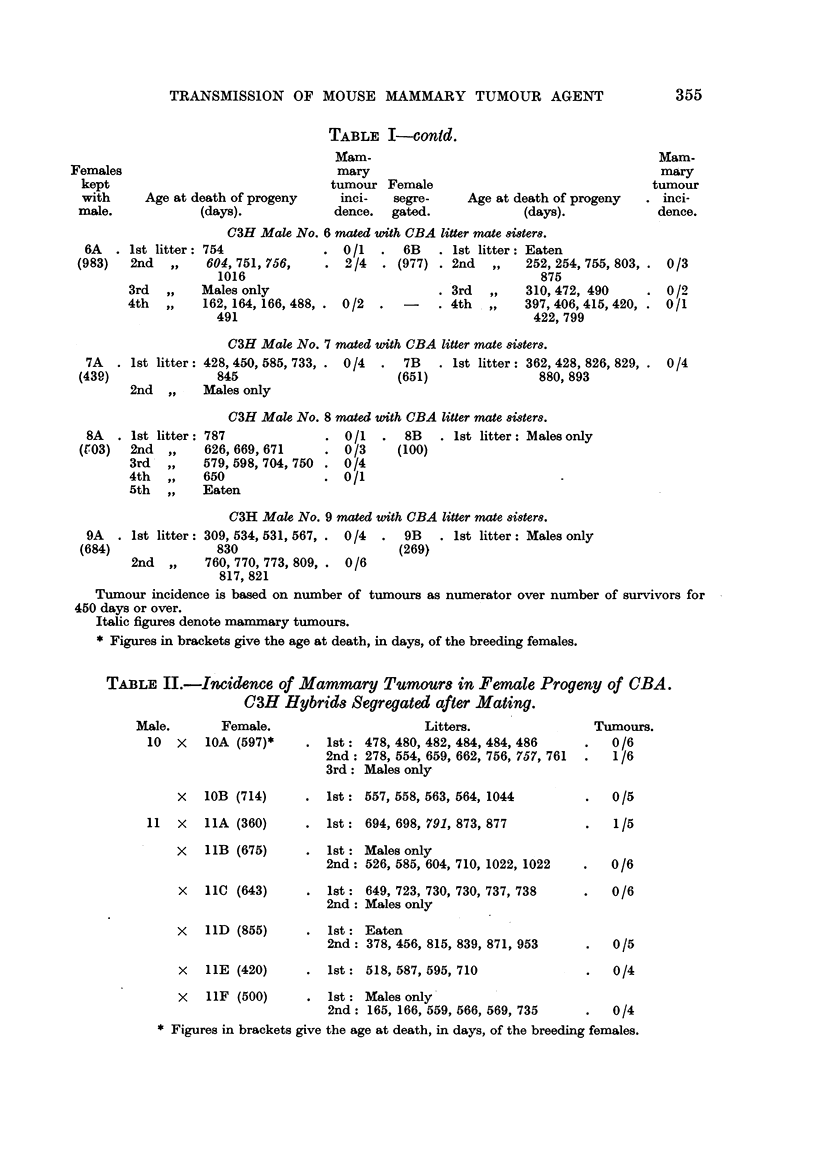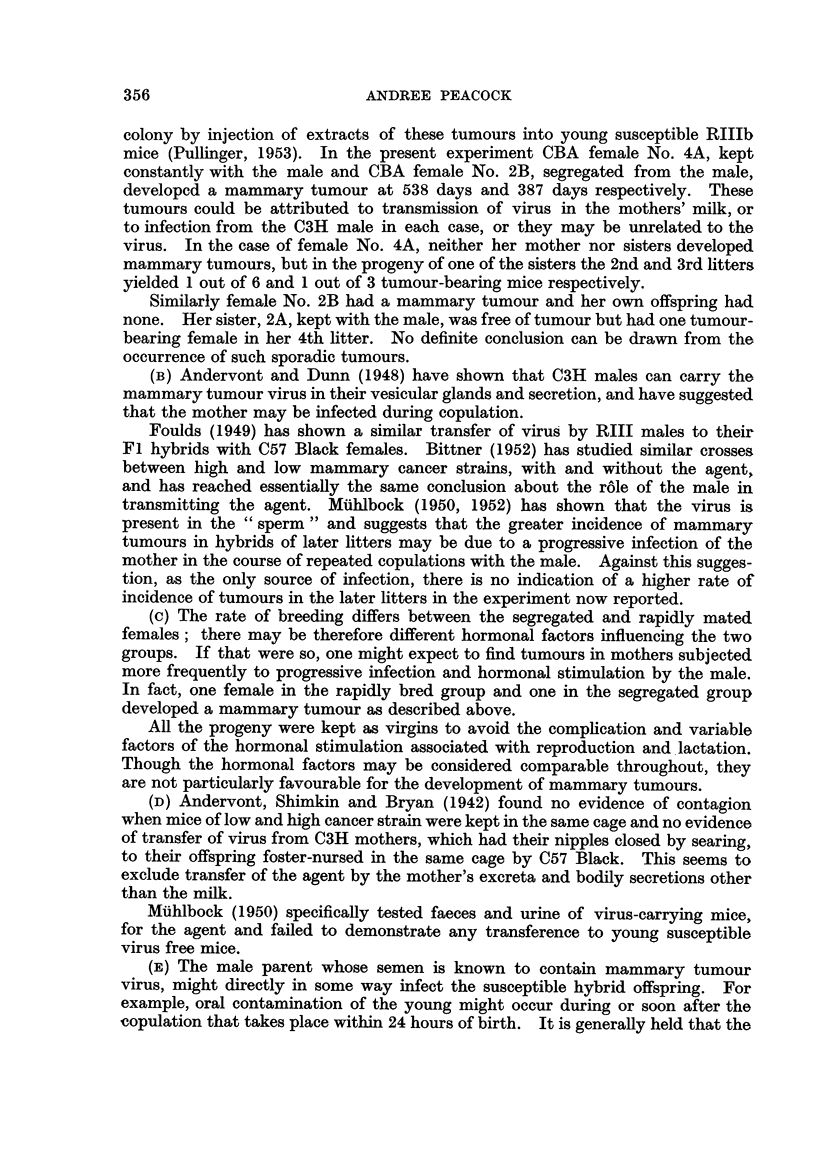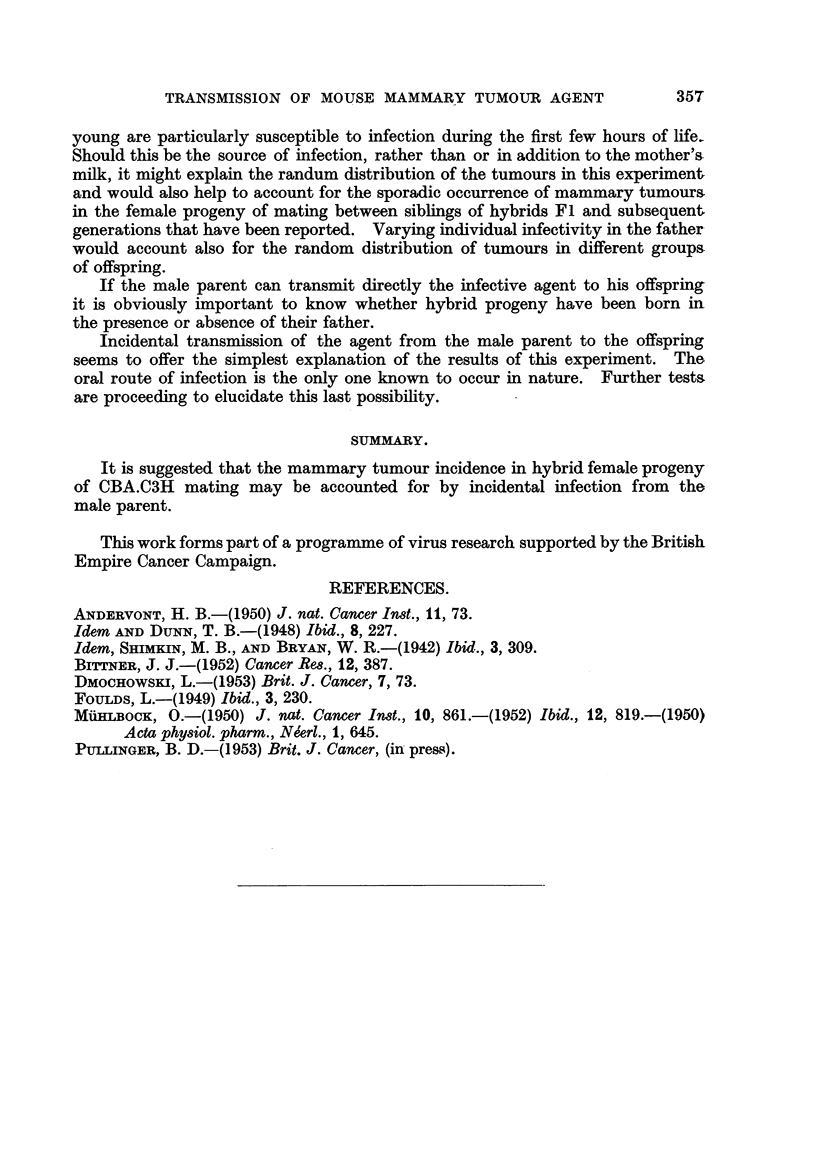# A Possible Mode of Transmission of the Mouse Mammary Tumour Agent by the Male Parent

**DOI:** 10.1038/bjc.1953.33

**Published:** 1953-09

**Authors:** Andree Peacock


### A POSSIBLE MODE OF TRANSMISSION OF THE MOUSE MAMMARY TUMOUR AGENT BY THE MALE PARENT.

ANDREE PEACOCK.*

*From the Cancer Research Department, Royal Beatson Memorial Hospital, Glasgow.*

Received for publication July 11, 1953.

IN the course of establishing a stock of hybrids between CBA (low cancer strain) females and C3H (high cancer strain) males, it was observed that the female offspring of CBA-C3H matings showed a higher incidence of mammary tumours than the CBA pure line : 20 mammary tumours in 61 hybrid mice that survived for more than 250 days when raised in the same cage with the father. The tumours were observed in mice between 259 and 973 days of age. This is in keeping with the observation of other workers using hybrids of low mammary cancer strain females and high mammary cancer strain males.

In this laboratory, CBA mice have been used in a number of experiments, but no large stock of untreated animals has been kept to check the natural incidence of mammary tumours. However, this cannot be high as amongst 50 control virgin CBA mice which died within the age of 12 to 25 months, no mammary tumours were seen, while in a group of 12 breeding females, 2 died with a mammary tumour, one at 565 days and the other at 777 days. In addition, sporadic mammary tumours have occurred in the breeding colony, that cannot be assessed statistically, because the breeders were often killed after producing several litters.

Dmochowski (1953) has recently reported the results of his own extensive experiments, and reviewed the work of others in this field.

It is known from the work of Andervont and Dunn (1948) that C3H males harbour the mammary tumour agent in the seminal vesicles, better called the vesicular glands, as they do not normally contain spermatozoa, and it has been suggested by Andervont (1950) and several subsequent investigators that the male of the higher cancer strain is responsible for infecting its female offspring indirectly by infecting the mother.

Several authors consider the possibility of direct infection of the embryo by the father, but dismiss it as against the evidence of all experiments of foster-nursing of high cancer strain mice by low cancer strain mothers. Direct infection of newly born offspring by their fathers does not seem to have been seriously considered.

The following experiment was carried out to test this possibility.

### EXPERIMENTAL.

### *CBA.C3H Hybrids.*

A C3H male was mated to two CBA female litter mates. One female remained constantly with the male throughout her breeding life ; the other female was removed to a sterilised box as soon as pregnancy was noted, and remained there

* Working under a Grant from the British Empire Cancer Campaign.

until her litter was weaned. She was then returned to her breeding box and the whole cycle was repeated. After weaning, the male offspring were killed and the females were kept as virgins, by litters, till death or appearance of mammary tumours. The temperature of the animal house was about 72° F. and the mice were kept on a balanced diet of commercial food pellets and water *ad libidum.*

Eventually the hybrids formed the following groups.

*Group I* consists of the offspring of males No. 2 to 7, mated each to 2 sisters, as originally planned.

*Group II* consists of the offspring of 3 sisters mated to male No. 1. Of the original 2 sisters, the segregated one died after her first litter, and was replaced by a third sister which was also segregated for birth and rearing of her first four litters. She was left with the male for the birth of her fifth litter (Table I.)

*Group III* consists of the offspring of 2 females, each permanently in the cage with males No. 8 and 9 respectively. The paired segregated sisters of each died early in the experiment, each after bearing a male litter.

*Group IV* consists of offspring of segregated females which were mated to make up a requisite number of mice in a given time. Two sisters were mated to male No. 10. Two sets of unrelated sisters were mated to male No 11. (Table II)

Ideally all the segregated females should have been sisters of the female left with the male, but this was not always possible.

The result of the experiment is summarised below :

| | |
|---|---|
| CBA<br>C3H. (mothers kept with male) | 11 mammary tumours out of 82 hybrids. |
| CBA.C3H<br>(mothers segregated from male) | 2 ,, ,, ,, ,, 84 ,, |

The incidence is expressed as the number of mice bearing mammary tumours as numerators over the number of survivors aged 450 days (age of youngest tumour-bearing mouse in this series) as denominators.

All these mammary tumours appearing in the hybrids were confirmed histologically by Dr. P. R. Peacock and were adenomata or adenocarcinomata of the type characteristic of those associated with the presence of the milk factor.

It was also noted at post mortem examination that many of the hybrids had cystic ovaries and enlarged uterine horns, the latter on histological examination showing varying degrees of cystic endometriosis.

*Fate of the CBA females mated with C3H males.*

Nine females were kept constantly with the male during the experiment ; one (4A) developed a mammary tumour and was killed at 538 days.

Seventeen females were repeatedly mated and segregated early in pregnancy ; one (2B) developed a mammary tumour and was killed at 387 days.

After the requisite number of offspring had been obtained, the mothers were segregated and kept for observation until the end of their lives (Tables I and II).

### DISCUSSION.

Although the number of animals is small, the incidence of approximately 5 times as many mammary tumours in these hybrids kept with their father as compared with those segregated from the same father, suggests some form of direct transmission from father to offspring. However, several factors have to be taken into consideration.

(A) Some of the CBA females may carry the agent and transmit it to their offspring. The presence of the virus has been shown in 2 tumours of the CBA

TABLE I.—*Incidence of Mammary Tumours in the Offspring of CBA Female Litter Mates Mated to a Common C3H Male.*

| Females kept with male. | | Age at death of progeny (days). | Mammary tumour incidence. | Female segregated. | | Age at death of progeny (days). | Mammary tumour incidence. |
|---|---|---|---|---|---|---|---|
| *C3H Male No.* 1 *mated with CBA litter mate sisters.* | | | | | | | |
| 1A | 1st litter: | 158, 177, 190 | — | 1B | 1st litter: | 571, 733, 993, 999 | 0/4 |
| (534)* | 2nd ,, | Males only | | (187) | | | |
| | 3rd ,, | 568, 731, 861 | o/2 | | | | |
| | 4th ,, | 557, 562, 563, 568 | 0/4 | | | | |
| | 5th ,, | 460, 780, 857 | 0/3 | | | | |
| | 6th ,, | Males only | | | | | |
| 1C | 5th ,, | *511*, 641, 651 | 1/3 | 1C | 1st litter: | Eaten | |
| | | | | (563) | 2nd ,, | ,, | |
| | | | | | 3rd ,, | 687 | 0/1 |
| | | | | | 4th ,, | Eaten | |
| *C3H Male No.* 2 *mated with CBA litter mate sisters.* | | | | | | | |
| 2A | 1st litter: | Died | — | 2B | 1st litter; | 569, 881 | 0/2 |
| (692) | 2nd ,, | Eaten | — | *387* | 2nd ,, | Males only | |
| | 3rd ,, | Eaten | — | | 3rd ,, | 718, 750, 928 | 0/3 |
| | 4th | 398, 410, 419, *843* | 1/1 | | | | |
| *C3H Male No.* 3 *mated with CBA litter mate sisters.* | | | | | | | |
| 3A | 1st litter: | Eaten | — | 3B | 1st litter: | 330, 755, 961, 1004 | 0/3 |
| (573) | 2nd ,, | 416, 557, 671, *678*, 687 733 | 1/5 | (477) | 2nd ,, | 595, 605, 608 | 0/3 |
| | 3rd ,, | *596*, 780, 836 | 1/3 | — | 3rd ,, | Males only | |
| | 4th ,, | Males only | — | — | 4th ,, | ,, ,, | |
| | 5th ,, | 767, 931 | 0/2 | | | | |
| | 6th ,, | Males only | | | | | |
| | 7th ,, | 400, 408, 409, 414, 416 | | | | | |
| *C3H Male No.* 4 *mated with CBA litter mate sisters.* | | | | | | | |
| 4A | 1st litter: | 1053 | 0/1 | 4B | 1st litter: | 684, 941 | 0/2 |
| *(538)* | 2nd ,, | 36, 37 | | (599) | 2nd ,, | 265, 265, 705, 812, 817, 832, 877 | 0/5 |
| | 3rd ,, | 641, 643, 645 | 0/3 | — | 3rd ,, | 645, 797, 811, 812 | 0/4 |
| | 4th ,, | 438, 655, 777, 798 | 0/3 | | | | |
| *C3H Male No.* 5 *mated with CBA litter mate sisters.* | | | | | | | |
| 5A | 1st litter: | *482* | 1/1 | 5B | 1st litter: | Eaten | |
| (725)* | 2nd ,, | 598, 600, 600, 602, *722* | 1/5 | (453) | 2nd ,, | ,, | |
| | 3rd ,, | *718*, 747, *795* | 2/3 | — | 3rd ,, | 433, 441, 444, 444, 447 | |
| | 4th ,, | Males only | | | | | |
| | 5th ,, | 576, 582, 582, 588, 900, 905, 938 | 0/7 0/2 | | | | |
| | 6th ,, | 588, 767 | | | | | |
| | 7th ,, | *500*, 556, 801 | 1/3 | | | | |

TABLE I—*contd.*

| Females kept with male. | | Age at death of progeny (days). | Mammary tumour incidence. | Female segregated. | | Age at death of progeny (days). | Mammary tumour incidence. |
|---|---|---|---|---|---|---|---|
| | | *C3H Male No.* 6 *mated with CBA litter mate sisters.* | | | | | |
| 6A (983) | 1st litter: | 754 | 0/1 | 6B (977) | 1st litter: | Eaten | |
| | 2nd „ | *604*, 751, *756*, 1016 | 2/4 | | 2nd „ | 252, 254, 755, 803, 875 | 0/3 |
| | 3rd „ | Males only | | | 3rd „ | 310, 472, 490 | 0/2 |
| | 4th „ | 162, 164, 166, 488, 491 | 0/2 | — | 4th „ | 397, 406, 415, 420, 422, 799 | 0/1 |
| | | *C3H Male No.* 7 *mated with CBA litter mate sisters.* | | | | | |
| 7A (439) | 1st litter: | 428, 450, 585, 733, 845 | 0/4 | 7B (651) | 1st litter: | 362, 428, 826, 829, 880, 893 | 0/4 |
| | 2nd „ | Males only | | | | | |
| | | *C3H Male No.* 8 *mated with CBA litter mate sisters.* | | | | | |
| 8A (503) | 1st litter: | 787 | 0/1 | 8B (100) | 1st litter: | Males only | |
| | 2nd „ | 626, 669, 671 | 0/3 | | | | |
| | 3rd „ | 579, 598, 704, 750 | 0/4 | | | | |
| | 4th „ | 650 | 0/1 | | | | |
| | 5th „ | Eaten | | | | | |
| | | C3H *Male No.* 9 *mated with CBA litter mate sisters.* | | | | | |
| 9A (684) | 1st litter: | 309, 534, 531, 567, 830 | 0/4 | 9B (269) | 1st litter: | Males only | |
| | 2nd „ | 760, 770, 773, 809, 817, 821 | 0/6 | | | | |

Tumour incidence is based on number of tumours as numerator over number of survivors for 450 days or over.

Italic figures denote mammary tumours.

* Figures in brackets give the age at death, in days, of the breeding females.

TABLE II.—*Incidence of Mammary Tumours in Female Progeny of CBA. C3H Hybrids Segregated after Mating.*

| Male. | | Female. | | Litters. | Tumours. |
|---|---|---|---|---|---|
| 10 | × | 10A (597)* | 1st: | 478, 480, 482, 484, 484, 486 | 0/6 |
| | | | 2nd: | 278, 554, 659, 662, 756, *757*, 761 | 1/6 |
| | | | 3rd: | Males only | |
| | × | 10B (714) | 1st: | 557, 558, 563, 564, 1044 | 0/5 |
| 11 | × | 11A (360) | 1st: | 694, 698, *791*, 873, 877 | 1/5 |
| | × | 11B (675) | 1st: | Males only | |
| | | | 2nd: | 526, 585, 604, 710, 1022, 1022 | 0/6 |
| | × | 11C (643) | 1st: | 649, 723, 730, 730, 737, 738 | 0/6 |
| | | | 2nd: | Males only | |
| | × | 11D (855) | 1st: | Eaten | |
| | | | 2nd: | 378, 456, 815, 839, 871, 953 | 0/5 |
| | × | 11E (420) | 1st: | 518, 587, 595, 710 | 0/4 |
| | × | 11F (500) | 1st: | Males only | |
| | | | 2nd: | 165, 166, 559, 566, 569, 735 | 0/4 |

* Figures in brackets give the age at death, in days, of the breeding females.

colony by injection of extracts of these tumours into young susceptible RIIIb mice (Pullinger, 1953). In the present experiment CBA female No. 4A, kept constantly with the male and CBA female No. 2B, segregated from the male, developed a mammary tumour at 538 days and 387 days respectively. These tumours could be attributed to transmission of virus in the mothers' milk, or to infection from the C3H male in each case, or they may be unrelated to the virus. In the case of female No. 4A, neither her mother nor sisters developed mammary tumours, but in the progeny of one of the sisters the 2nd and 3rd litters yielded 1 out of 6 and 1 out of 3 tumour-bearing mice respectively.

Similarly female No. 2B had a mammary tumour and her own offspring had none. Her sister, 2A, kept with the male, was free of tumour but had one tumour-bearing female in her 4th litter. No definite conclusion can be drawn from the occurrence of such sporadic tumours.

(B) Andervont and Dunn (1948) have shown that C3H males can carry the mammary tumour virus in their vesicular glands and secretion, and have suggested that the mother may be infected during copulation.

Foulds (1949) has shown a similar transfer of virus by RIII males to their F1 hybrids with C57 Black females. Bittner (1952) has studied similar crosses between high and low mammary cancer strains, with and without the agent, and has reached essentially the same conclusion about the rôle of the male in transmitting the agent. Mühlbock (1950, 1952) has shown that the virus is present in the " sperm " and suggests that the greater incidence of mammary tumours in hybrids of later litters may be due to a progressive infection of the mother in the course of repeated copulations with the male. Against this suggestion, as the only source of infection, there is no indication of a higher rate of incidence of tumours in the later litters in the experiment now reported.

(C) The rate of breeding differs between the segregated and rapidly mated females ; there may be therefore different hormonal factors influencing the two groups. If that were so, one might expect to find tumours in mothers subjected more frequently to progressive infection and hormonal stimulation by the male. In fact, one female in the rapidly bred group and one in the segregated group developed a mammary tumour as described above.

All the progeny were kept as virgins to avoid the complication and variable factors of the hormonal stimulation associated with reproduction and lactation. Though the hormonal factors may be considered comparable throughout, they are not particularly favourable for the development of mammary tumours.

(D) Andervont, Shimkin and Bryan (1942) found no evidence of contagion when mice of low and high cancer strain were kept in the same cage and no evidence of transfer of virus from C3H mothers, which had their nipples closed by searing, to their offspring foster-nursed in the same cage by C57 Black. This seems to exclude transfer of the agent by the mother's excreta and bodily secretions other than the milk.

Mühlbock (1950) specifically tested faeces and urine of virus-carrying mice, for the agent and failed to demonstrate any transference to young susceptible virus free mice.

(E) The male parent whose semen is known to contain mammary tumour virus, might directly in some way infect the susceptible hybrid offspring. For example, oral contamination of the young might occur during or soon after the copulation that takes place within 24 hours of birth. It is generally held that the young are particularly susceptible to infection during the first few hours of life. Should this be the source of infection, rather than or in addition to the mother's milk, it might explain the randum distribution of the tumours in this experiment and would also help to account for the sporadic occurrence of mammary tumours in the female progeny of mating between siblings of hybrids F1 and subsequent generations that have been reported. Varying individual infectivity in the father would account also for the random distribution of tumours in different groups of offspring.

If the male parent can transmit directly the infective agent to his offspring it is obviously important to know whether hybrid progeny have been born in the presence or absence of their father.

Incidental transmission of the agent from the male parent to the offspring seems to offer the simplest explanation of the results of this experiment. The oral route of infection is the only one known to occur in nature. Further tests are proceeding to elucidate this last possibility.

### SUMMARY.

It is suggested that the mammary tumour incidence in hybrid female progeny of CBA.C3H mating may be accounted for by incidental infection from the male parent.

This work forms part of a programme of virus research supported by the British Empire Cancer Campaign.

### REFERENCES.

Andervont, H. B.—(1950) *J. nat. Cancer Inst.*, **11**, 73.
*Idem* and Dunn, T. B.—(1948) *Ibid.*, **8**, 227.
*Idem*, Shimkin, M. B., and Bryan, W. R.—(1942) *Ibid.*, **3**, 309.
Bittner, J. J.—(1952) *Cancer Res.*, **12**, 387.
Dmochowski, L.—(1953) *Brit. J. Cancer*, **7**, 73.
Foulds, L.—(1949) *Ibid.*, **3**, 230.
Mühlbock, O.—(1950) *J. nat. Cancer Inst.*, **10**, 861.—(1952) *Ibid.*, **12**, 819.—(1950) *Acta physiol. pharm., Néerl.*, **1**, 645.
Pullinger, B. D.—(1953) *Brit. J. Cancer*, (in press).